# Structural biology of laminins

**DOI:** 10.1042/EBC20180075

**Published:** 2019-05-15

**Authors:** Erhard Hohenester

**Affiliations:** Department of Life Sciences, Imperial College London, London SW7 2AZ, U.K.

**Keywords:** cell adhesion, crystallography, extracellular matrix

## Abstract

Laminins are large cell-adhesive glycoproteins that are required for the formation and function of basement membranes in all animals. Structural studies by electron microscopy in the early 1980s revealed a cross-shaped molecule, which subsequently was shown to consist of three distinct polypeptide chains. Crystallographic studies since the mid-1990s have added atomic detail to all parts of the laminin heterotrimer. The three short arms of the cross are made up of continuous arrays of disulphide-rich domains. The globular domains at the tips of the short arms mediate laminin polymerization; the surface regions involved in this process have been identified by structure-based mutagenesis. The long arm of the cross is an α-helical coiled coil of all three chains, terminating in a cell-adhesive globular region. The molecular basis of cell adhesion to laminins has been revealed by recent structures of heterotrimeric integrin-binding fragments and of a laminin fragment bound to the carbohydrate modification of dystroglycan. The structural characterization of the laminin molecule is essentially complete, but we still have to find ways of imaging native laminin polymers at molecular resolution.

## Introduction

About 40 years ago, two laboratories independently purified a large glycoprotein from the extracellular matrix produced by mouse tumour cells [1,2]. Antibodies raised against this glycoprotein reacted with basement membranes (also known as basal laminae) in mouse tissues, prompting Rupert Timpl and colleagues to name the new protein laminin. We now know that mammals have at least 15 laminin isoforms (invertebrates have only two) and that laminins are essential: in their absence, basement membranes do not form and embryo development is arrested at an early stage [3–6]. Similarly to fibronectin (another important glycoprotein of the extracellular matrix), laminins form networks and bind to cellular receptors. Defective cell adhesion to laminins results in severe human diseases, such as muscular dystrophies and skin blistering disorders [7,8]. There are many reviews on laminin structure and function, but none that takes into account the recent surge of crystal structures. Here, I will discuss the new structures and place them in the context of four decades of laminin research. As will become clear, we have reached a near-complete description of laminin structure at the level of the heterotrimer, but we are still some way from understanding the networks that exist in basement membranes.

**Figure 1:**
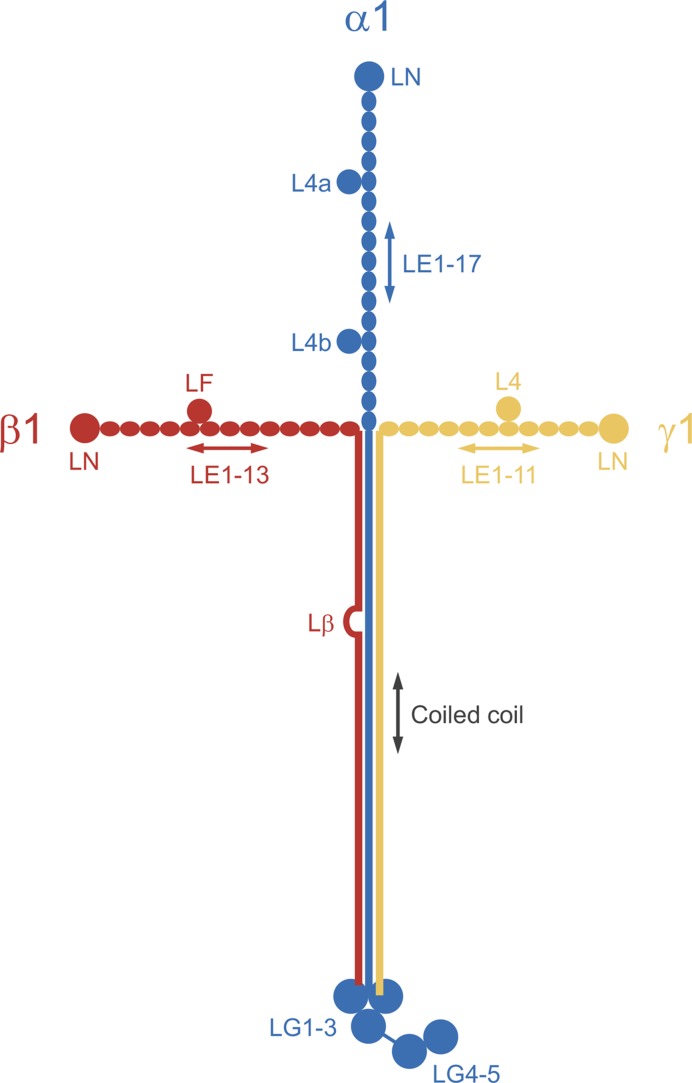
Schematic drawing of the laminin-111 heterotrimer The three short arms of the cross-shaped laminin molecule correspond to the N-terminal regions of the three chains, while the long arm is an α-helical coiled coil of all three chains. The mature polypeptide chains of mouse laminin-111 have 3059 (α1 chain), 1765 (β1 chain), and 1574 (γ1 chain) residues. The calculated molecular mass of the heterotrimer is 705 kDa, but the experimental mass is close to 900 kDa due to extensive glycosylation. Lβ, cysteine-rich interruption of the heptad repeats in the β1 chain.

Laminins are heterotrimers consisting of one α, one β, and one γ chain. Mammalian genomes encode five α chains, four β chains, and three γ chains; only 15 of the 60 possible heterotrimers have been confirmed biochemically [9,10]. The laminin originally purified from mouse tumour matrix is the α1β1γ1 heterotrimer, now called laminin-111 (M_r_ ∼900 kDa). The three laminin-111 chains associate via a long α-helical coiled coil (Figure 1). The coiled coil extends all the way to the C-terminus in the β1 and γ1 chains, whereas in the α1 chain it is followed by five laminin G-like (LG) domains. The chains are covalently linked by disulphide bonds: three at the start of the coiled coil and another one linking the C-termini of the β1 and γ1 chains. The N-terminal regions preceding the coiled coil have a similar structure in all three chains: they consist of a globular laminin N-terminal (LN) domain followed by tandemly repeated laminin-type epidermal growth factor-like (LE) domains; in each chain, one or two globular laminin type IV (L4 and LF) domains are inserted into the array of LE domains. Other laminins have a similar architecture to laminin-111, except that some chains (α3A, α4, and γ2) are missing the LN domain and some or all of the LE repeats [9].

The first insights into the structure of laminin-111 were obtained by using rotary shadowing electron microscopy, which revealed four arms: a long arm (∼77 nm in length) terminating in a pronounced globule (the ‘G domain’ from which the LG domains derive their name), and three shorter arms (∼34 nm in length) terminating in a pair of smaller globules [11]. Although a range of shapes was seen in the micrographs, only a cross with roughly perpendicular arms has entered the literature. This is somewhat unfortunate as the structure of laminin within a basement membrane is more likely to resemble a three-spoked umbrella instead of a cross (see below). The cDNA sequencing of laminin chains in the 1980s allowed shape to be related to sequence, resulting in a laminin-111 model that has required remarkably little revision in the years since [12,13]. Functionally, there is a clear division of labor between the four arms of laminin heterotrimers: the N-termini of the short arms mediate laminin polymerization, whereas the C-terminus of the long arm mediates interactions with the cell surface [5]. As we will see, crystal structures of recombinant laminin fragments have made significant contributions to this understanding of laminin function.

## Laminin short-arm structures

**Figure 2:**
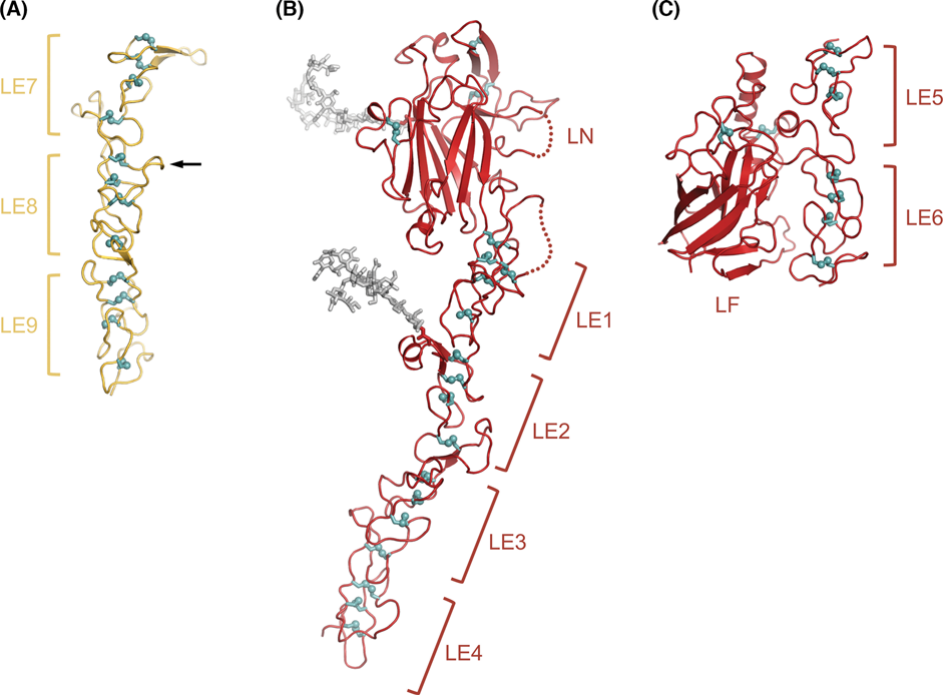
Crystal structures of laminin short arm fragments (**A**) Laminin γ1 LE7-9 structure, showing the characteristic rod-like arrangement of consecutive LE domains [14]. Disulphide bonds are shown in teal with sulphur atoms represented as spheres. The arrow indicates the location of the nidogen binding site. (**B**) Laminin β1 LN-LE1-4 structure, showing the LN domain attached to the tip of the LE1-4 rod [17]. Two *N*-linked glycosylation sites are shown with biantennary glycan structures (light gray sticks). The glycan on the LN domain is attached to the ‘back’ face (see text). (**C**) Laminin β2 LE5-LF-LE6 structure [20], showing that the LF domain does not interrupt the rod-like arrangement of LE domains.

Structural studies of laminin entered the atomic age in 1996, when Stetefeld and colleagues [14] determined the crystal structures of LE domains 7–9 of the γ1 chain (Table 1). They were interested in this region because γ1 LE8 contains a high-affinity binding site for nidogen, another important basement membrane protein. The rod-like laminin γ1 LE7-9 structure lacks a defined hydrophobic core; instead, the LE fold is stabilized by four disulphide bonds that are arranged like the rungs of a ladder (Figure 2A). The nidogen binding site is formed by a protruding loop in LE8, which in a later structure from Timothy Springer’s laboratory [15] was shown to insert into the center of a β-propeller domain in nidogen. The disulphide bonding pattern established by the γ1 LE7-9 structure (i.e., eight cysteines linked 1–3, 2–4, 5–6, and 7–8) was subsequently observed in all other LE domain-containing structures; an alternative pattern proposed on the basis of mass spectrometry experiments [16] could not be verified.

**Table 1:** Crystal structures of laminin fragments

Laminin region (+ ligand)	PDB code	Resolution (Å)	Reference
γ1 LE7-9	1KLO	2.1	[14]
α2 LG5	1QU0	2.4	[62]
α2 LG4-5	1DYK	2.0	[63]
γ1 LE7-9 + nidogen G3	1NPE	2.3	[15]
α1 LG4-5	2JD4	1.9	[66]
α2 LG1-3	2WJS	2.8	[68]
α5 LN-LE1-2	2Y38	2.9	[18]
β1 LN-LE1-4	4AQS	3.1	[17]
γ1 LN-LE1-2	4AQT	3.2	[17]
α2 L4b	4YEP	1.2	[19]
α2 LG4-5 + matriglycan oligosaccharide	5IK5, 5IK8	1.4, 2.0	[65]
β2 LE5-LF-LE6	5LF2	1.9	[20]
α1β1γ1 integrin binding fragment	5MC9	2.1	[35]
α5β1γ1 integrin binding fragment	5XAU	1.8	[36]

The LN domain structure was elucidated by my laboratory in 2011 [17,18]. Because it is not possible to produce LN domains in isolation, we crystallized longer fragments containing the LN domain and a small number of LE domains: α5 LN-LE1-2, β1 LN-LE1-4, and γ1 LN-LE1-2. The three structures are quite similar and only the β1 LN-LE1-4 structure is shown in Figure 2B. The LE1-4 portion of the laminin β1 chain conforms to the principles established by the γ1 LE7-9 structure. The LN domain is an antiparallel β-sandwich containing a jelly roll motif; the closest structural relatives are bacterial galactose-binding domains. The ‘top’ of the β-sandwich (i.e., the edge farthest from the LE1-4 rod) is sealed off by a disulphide-bonded hairpin. One face of the β-sandwich (the ‘back’) carries an *N*-linked glycan in all laminin LN domain, while the front face is free of glycans in all LN domains. This observation contributed to the identification of surface regions involved in laminin polymerization (see below).

The functions of the internal globular domains of laminin short arms, L4 and LF, are unknown. The structure of a representative L4 domain (α2 L4b) was reported in 2015 and revealed another antiparallel β-sandwich fold [19]. Biochemical experiments showed that the L4b domain is inserted into LE14 of the laminin α2 chain without disrupting the characteristic disulphide bonding pattern, suggesting that the L4 domains are located peripherally on a continuous LE tandem array (confirmed by an unpublished structure from my laboratory). We determined the structure of the β2 LF domain in the context of the flanking LE5 and LE6 domains [20] (Figure 2C). The structure revealed that the LF domain is distantly related to the L4 domain; this relationship had not been evident from sequence comparison. The LE5 domain lacks the 7–8 disulphide bond, allowing the LF domain to insert between LE5 and LE6 without disrupting the typical rod-like interaction of the two domains. Thus, also the LF domain is located peripherally. The original laminin-111 model, as well as all subsequent variations of it, depicts the L4 and LF domains as globules within laminin’s short arms. The diagram in Figure 1 has been modified to emphasize the continuous nature of the LE arrays in all three short arms. The arms are not completely straight, however. A solution X-ray scattering study of the entire γ1 short arm revealed a pronounced kink roughly at the position of the L4 domain [21] and the β2 short arm also appears to be curved [20].

## Molecular mechanism of laminin polymerization

**Figure 3:**
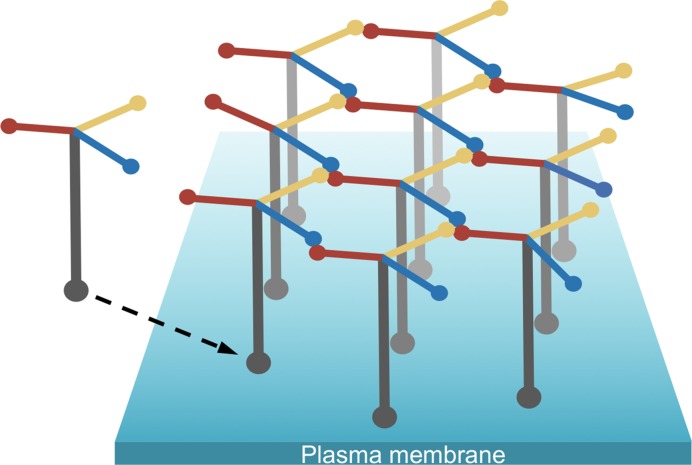
Laminin polymerization at the cell surface Schematic drawing of a cell-associated laminin-111 polymer. The tips of three short arms form the ternary nodes of the laminin polymer, and the tip of the long arm interacts with various receptors embedded in the plasma membrane [5].

Laminin-111 purified from mouse tumour matrix can be polymerized by heating to 37°C and depolymerized by cooling to 4°C [22]. The mechanism of laminin-111 polymerization was worked out before the recombinant era, by using structurally defined laminin-111 fragments obtained by limited proteolysis. According to Peter Yurchenco’s seminal ‘three-arm interaction model’, the N-terminal regions of one α1, one β1, and one γ1 chain combine to form the nodes of a quasi-hexagonal network [23]. This model has been challenged [24], but there is now overwhelming evidence in its favor. First, experiments with truncated and chimeric full-length laminin heterotrimers have shown that only laminins with a full complement of LN domains (i.e., one α, one β, and one γ LN domain) polymerize *in vitro* and on cultured cells [25]. Second, polymerization of laminin heterotrimers lacking LN domains can be restored by chimeric proteins in which the missing LN domain is fused to nidogen (which creates an artifical short arm branching off at γ1 LE8) [26,27]. Third, biophysical studies with recombinant short arm tips have shown that the assembly of ternary αβγ nodes is a highly cooperative process, with only one detectable intermediate (a weak βγ dimer) [18,28]. Laminin polymerization *in vivo* occurs at the cell surface and requires not only the three short arms but also the cell-binding long arm, which helps to concentrate laminin at the cell surface [5]. The currently accepted model of a cell-anchored laminin polymer is shown in Figure 3. It is important to realize that this model is largely derived from *in vitro* experiments rather than from direct imaging of basement membranes. However, indirect support for an erect arrangement of laminin heterotrimers on the cell surface comes from super-resolution fluorescence microscopy of the glomerular basement membrane [29].

How do the three short arm tips combine to form the nodes of the laminin network? Experiments with chimeric proteins have narrowed down the regions involved in node formation to the LN domain and the first LE domain, at least for the β1 and γ1 arms [25,28]. Our structural analysis of LN domains revealed a markedly uneven distribution of glycosylation sites and surface conservation, which suggested that the front faces of LN domains are involved in protein–protein interactions [17,18]. Indeed, mutation of residues on the front face of the α5 LN domain abolished ternary node formation [18]. Missense mutations in patients with muscular dystrophy (α2 chain) and renal Pierson syndrome (β2 chain), which weaken the laminin polymer, also map to the front face of the LN domain [30]. A structure of a laminin network node is needed, but crystallization of reconstituted nodes has proved to be challenging. Even a low-resolution structure of a ternary node, for example by electron microscopy, would be extremely informative, given that we already know the atomic structures of individual short arm tips.

It is currently not known whether laminins lacking a full complement of short arms form networks similar to that of laminin-111. Laminins with truncated short arms, such as the skin-specific laminin-332, are evidently incorporated into functional basement membranes, but this may be due to heterotypic interactions with other basement membrane components rather than homotypic laminin polymerization. A network consisting predominantly of laminin-332 (but, perhaps significantly, also containing laminin α5 chains) was isolated from dissociated human dermis and visualized by electron microscopy as a finely grained mesh; the resolution was not sufficient to define the molecular structure of the polymer, however [31]. Further biochemical examination of laminin-332 interactions is warranted.

## The α-helical coiled coil of laminin

**Figure 4:**
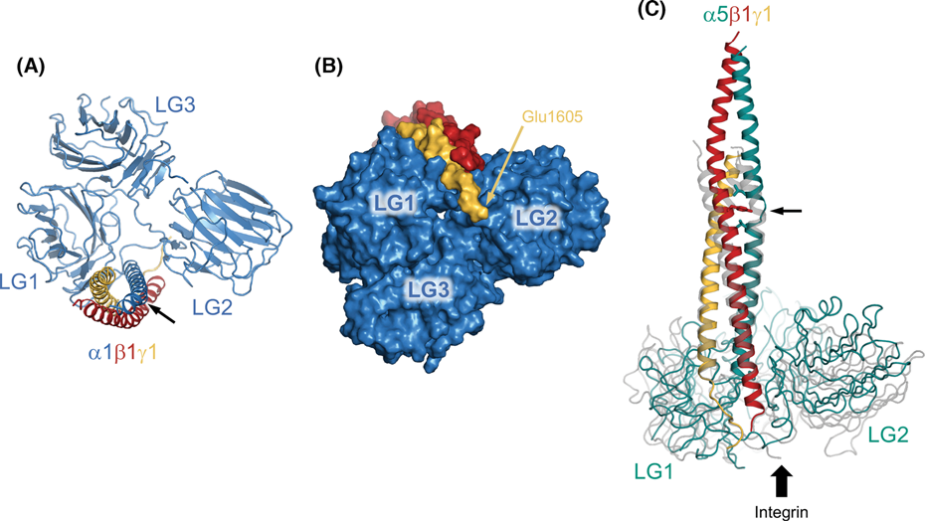
Crystal structures of heterotrimeric integrin-binding laminin fragments Integrin binding requires the C-terminal portion of the heterotrimeric coiled coil, together with domains LG1-3 of the laminin α chain [5]. (**A**) Structure of the laminin-111 mini-E8 fragment [35] viewed down the α-helical coiled coil, i.e., from N- to C-terminus. This view shows the ‘top’ surface of the LG1-3 triangle. The arrow indicates a kink in the α1 helix introduced by Pro2095. (**B**) Space-filling representation of the mini-E8 structure showing the ‘bottom’ surface of the LG1-3 triangle. The position of the critical integrin-binding E1605 residue (mouse γ1 chain) is indicated. The views in (A) and (B) are related by a 180° rotation about the horizontal axis. (**C**) Structure of the laminin-511 E8-like fragment [36] viewed from the side, superimposed onto the corresponding laminin-111 structure (in transparent gray). The arrow indicates an interruption to the regular heptad repeats in the α5 chain (see text). Integrin binding to the γ1 chain tail is indicated by the block arrow.

The presence of an α-helical coiled coil in the long arm of laminin-111 was first suggested in the mid-1980s based on partial sequences of the β1 and γ1 chains [32] and on biophysical experiments with a proteolytic laminin-111 fragment termed E8 [33]. The heterotrimeric nature of the coiled coil was established a few years later, when the α1 chain sequence became available [12] and the E8 fragment was shown to consist of the C-terminal third of the heterotrimeric coiled coil and domains LG1-3 of the α1 chain [34]. Recently, my laboratory determined the crystal structure of a shortened E8 fragment (mini-E8) [35] and Kiyotoshi Sekiguchi’s laboratory reported an analogous structure from laminin-511 [36]. In our mini-E8 structure (Figure 4A,B), the last six heptad repeats of the α1, β1, and γ1 helices are aligned regularly, but only the β1 and γ1 helices display the gentle curvature that is characteristic of coiled coils; the α1 helix has a pronounced kink at Pro2095 and then runs straight for the last eight turns. These features may relate to the stepwise assembly mechanism of the coiled coil, in which a dimeric β1γ1 coiled coil is proposed to form first, followed by insertion of the α1 chain [37]. In the structure of the laminin-511 E8-like fragment (Figure 4C), the coiled coil has an imperfection between α5 residues Leu2700 and Val2705, where the α-helix is interrupted to accommodate Tyr1747 of the β1 chain. It is evident from sequence analysis that such deviations from a regular coiled coil are common throughout the long arm [13]. A recent study from Deborah Fass’s laboratory used chemical cross-linking and mass spectrometry to define the register changes within the long arm of laminin-111; both the clockwise arrangement of α1, β1 and γ1 (when viewed from the N-terminus) and the chain register in the mini-E8 structure were predicted correctly [38]. Apart from holding the three laminin chains together, the coiled coil has at least one other function: its central region contains a binding site for the basement membrane proteoglycan, agrin [39].

## The integrin binding site of laminins

An important early finding was that laminin-111 stimulates the outgrowth of neurites from cultured neurons [40,41]. The cellular receptor responsible for this activity was subsequently identified as a member of the integrin family and its binding site mapped to the E8 fragment [42–45]. We now know that four of the 24 mammalian integrins are laminin receptors, namely integrins α3β1, α6β1, α6β4 and α7β1 [46]. Laminin–integrin interactions are not only essential for embryo development and tissue function in animals but also support the long-term self-renewal of human stem cells in the laboratory [47,48]. Unlike the integrin binding site of fibronectin which can be reduced to a short peptide motif (the famous RGD sequence), the integrin binding site in laminin-111 strictly depends upon the native heterotrimeric structure of the E8 region [49]. All attempts to further narrow down the integrin binding site were unsuccessful, until Sekiguchi’s laboratory demonstrated in 2007 that a glutamic acid close to the C-terminus of the γ1 chain is strictly required for integrin binding [50]. The recent crystal structures of E8-like fragments [35,36] showed that the coiled coil is attached perpendicularly to a triangular arrangement of domains LG1-3 of the α chain, with the γ1 tail nestled between LG1 and LG2 on the more accessible ‘bottom’ surface of the LG1-3 triangle (Figure 4). Cross-linking experiments showed that the γ1 tail interacts with the metal ion-dependent adhesion site in the integrin β1 subunit [36]. Additional contacts are likely to be made by the LG1 and LG2 surfaces flanking the γ1 tail, which would explain why the integrin specificity of laminin heterotrimers is largely determined by the identity of the α chain [46]. An electron micrograph of integrin α6β1 bound to the laminin-511 E8 fragment indeed shows the integrin approaching the LG domains from the opposite direction as the coiled coil [36], but a high-resolution structure is needed to understand how specificity is achieved. Such a structure would also provide invaluable information on the conformational regulation of laminin-binding integrins, which appears to deviate from the prevailing model derived from integrins containing the β2 and β3 subunits [51]. Finally, the laminin-111 mini-E8 structure has laid to rest the notion that a linear sequence motif in the laminin α1 chain, IKVAV, is an active site for cell adhesion and neurite outgrowth [52]. The IKVAV motif is completely buried in the mini-E8 structure and the biological activities of IKVAV peptides, therefore, cannot reflect those of native laminin-111.

## LG domain structure and dystroglycan binding

Dystroglycan is a receptor on muscle and nerve cells that uses a unique carbohydrate modification to bind laminin heterotrimers containing the α1, α2, or α5 chain. The chemical structure of the laminin-binding carbohydrate was recently elucidated; we now know that laminins binds to a glucuronic acid-xylose (GlcA-Xyl) polysaccharide termed matriglycan, which is attached to the dystroglycan core protein via an unusual heptasaccharide linker [53–56]. The laminin–dystroglycan interaction is essential for muscle function: mutations that inactivate the enzymes involved in dystroglycan modification cause a group of muscular dystrophies known as the dystroglycanopathies [53–56]. Dystroglycan interacts primarly with the LG4 domains of the laminin α1, α2, and α5 chains, and it does so in a strictly Ca^2+^-dependent manner [57–60]. The laminin α2 chain is unique in having an additional dystroglycan binding site in the LG1-3 region [59,61]. The first LG domain structure (LG5 of the α2 chain) was determined 20 years ago in my laboratory and revealed an antiparallel β-sandwich related to the pentraxin fold [62]. An incompletely coordinated Ca^2+^ ion was identified at one edge of the β-sandwich. This observation led us to predict that the (then uncharacterized) carbohydrate modification of dystroglycan might complete the Ca^2+^ coordination sphere, thus accounting for the Ca^2+^-dependence of the interaction [62]. A subsequent structure of the V-shaped LG4-5 domain pair [63] visualized the dystroglycan-binding Ca^2+^ site in LG4, but further progress had to await the chemical characterization of the laminin-binding modification on dystroglycan.

**Figure 5:**
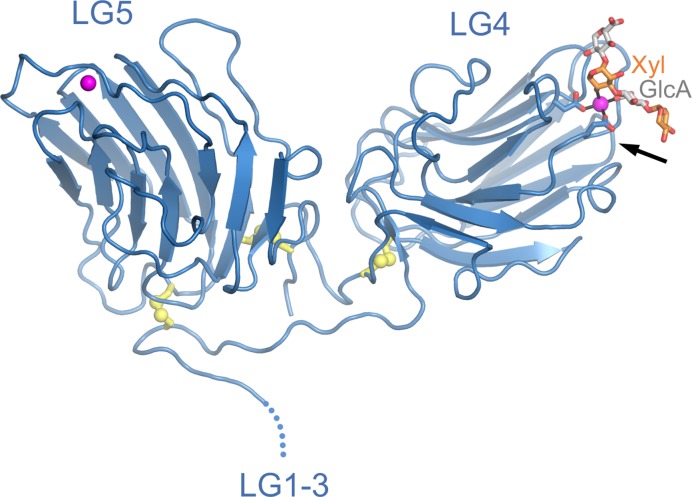
Crystal structure of laminin α2 LG4-5 bound to a matriglycan oligosaccharide Matriglycan is a GlcA-Xyl polysaccharide attached to the transmembrane protein dystroglycan [53–56]. A GlcA-Xyl oligosaccharide was synthesized enzymatically using LARGE and soaked into crystals of laminin α2 LG4-5 [65]. The Ca^2+^ ions in LG4 and LG5 are represented as magenta spheres. Disulphide bonds are shown in yellow with sulphur atoms represented as spheres. The oligosaccharide is shown in stick representation, with GlcA and Xyl carbon atoms in light gray and orange, respectively. The arrow indicates the location of heparin-binding residues in the corresponding α1 LG4-5 structure [66].

In 2012, Kevin Campbell’s laboratory reported that the laminin-binding polysaccharide (i.e., matriglycan) is synthesized by a bifunctional glycosyltransferase called LARGE [64]. This discovery made it possible to synthesize defined GlcA-Xyl oligosaccharides for biophysical and structural studies. By soaking such an oligosaccharide into crystals of laminin α2 LG4-5, we were able to obtain a high-resolution structure of a minimal laminin-matriglycan complex [65] (Figure 5). As predicted [62], the GlcA-Xyl oligosaccharide completes the Ca^2+^ coordination sphere in LG4: a single GlcA-Xyl unit straddles the Ca^2+^ ion, such that each sugar makes one bond to the Ca^2+^ ion. A longer matriglycan chain may additionally interact with basic residues in LG4, which are important for heparin binding (heparin is a highly sulphated version of the abundant glycosaminoglycan, heparan sulphate) [58,60,66,67]. The relative contributions of integrins, matriglycan, and heparan sulphate to laminin binding are likely to depend on cell type and laminin isoform. The LG1-3 and LG4-5 portions of laminin α chains are separated by a flexible linker, suggesting that integrin and matriglycan binding may not be mutually exclusive.

## Concluding remarks

The laminin molecule has been the subject of structural investigations for nearly 40 years, beginning with the first glimpses of its iconic shape and eventually progressing to atomic structures of all of its constituent domains. The era of discoveries appears to be over. There is still work to be done on the molecular mechanisms of laminin polymerization and integrin binding, but I expect these studies to refine rather than upset our current understanding. In writing this historical survey of laminin structure, I was struck by how much was worked out before recombinant technology started to make an impact on the field. Clearly, the early discoveries owed much to an abundant source of laminin-111 (the mouse tumour matrix), but they are also a testament to the protein chemistry skills of the pioneers. Is there still a frontier in the structural biology of laminins? The answer is an emphatic ‘yes’: no one has yet been able to image a cell-associated laminin network (let alone a native basement membrane) at a resolution that allows individual molecules to be distinguished. It should be possible to assemble laminin networks on artificial lipid bilayers and study these structures using modern electron microscopy techniques. Efforts should be made to improve procedures for extracting basement membranes from tissues and to investigate new ways of growing basement membranes in organoid cultures. The biggest challenge, however, may be to enthuse a new generation of structural biologists for what many consider a solved problem.

## Summary

- The structural characterization of the cross-shaped laminin heterotrimer is essentially complete.
- Biophysical studies support the ‘three-arm interaction model’ of laminin polymerization, but the molecular mechanism of ternary node formation remains to be determined.
- Crystal structures have revealed how the three chains of laminin combine to form a functional integrin binding site.
- A crystal structure has revealed how laminins recognize the glucuronic acid-xylose polysaccharide on dystroglycan.

## Abbreviations

GlcA: glucuronic acid
LE: laminin-type epidermal growth factor-like domain
LF/L4: laminin type IV domain
LG: laminin G-like domain
LN: laminin N-terminal domain
Xyl: xylose
